# Dr. Edward Gamaliel Janeway

**Published:** 1911-03

**Authors:** 


### OBITUARY

Dr. Edward Gamaliel Janeway died at his country home in Summit, N. J., February 10, 1911, aged 69 years. He was born near New Brunswick, N. J., August 31, 1841, and obtained his preliminary education in that state. He then entered the College of Physicians and Surgeons, New York, from which he took his medical degree in 1864, one year, 1862-1863, being spent as an acting medical cadet in the United States army hospital at Newark, N. J.

Since 1864 Dr. Janeway practised his profession continuously in the city of New York, holding many college and hospital appointments. He was curator of the College of Physicians and Surgeons from 1868 to 1872, professor of pathology and practical anatomy from 1872 to 1879, and professor of diseases of the mind and nervous system from 1881 to 1886; professor of medicine in Bellevue Hospital Medical College from 1886 to 1892, and dean of the University and Bellevue Hospital Medical College since 1908. From 1875 to 1882 he served also as Health Commissioner of New York.

At the time of his death he was consulting physician to the Presbyterian, Bellevue, St. Luke's, Mt. Sinai, St. Vincent's, French, and Woman's Hospitals, and the Hospital for Ruptured and Crippled. He was a member of the several local, state and national medical societies and was a director of the Russell Sage Institute of Pathology.

